# Review of Fiber Optic Sensors for Structural Fire Engineering

**DOI:** 10.3390/s19040877

**Published:** 2019-02-20

**Authors:** Yi Bao, Ying Huang, Matthew S. Hoehler, Genda Chen

**Affiliations:** 1 Department of Civil, Environmental and Ocean Engineering, Stevens Institute of Technology, 1 Castle Point Terrace, Hoboken, NJ 07030, USA; yi.bao@stevens.edu; 2 Department of Civil and Environmental Engineering, North Dakota State University, Fargo, ND 58105, USA; ying.huang@ndsu.edu; 3 National Fire Research Laboratory, National Institute of Standards and Technology, Gaithersburg, MD 20899, USA; matthew.hoehler@nist.gov; 4 Department of Civil, Architectural and Environmental Engineering, Missouri University of Science and Technology, Rolla, MO 65409, USA

**Keywords:** fiber optic sensors, high temperature, intelligent sensors, smart structure, structural fire engineering

## Abstract

Reliable and accurate measurements of temperature and strain in structures subjected to fire can be difficult to obtain using traditional sensing technologies based on electrical signals. Fiber optic sensors, which are based on light signals, solve many of the problems of monitoring structures in high temperature environments; however, they present their own challenges. This paper, which is intended for structural engineers new to fiber optic sensors, reviews various fiber optic sensors that have been used to make measurements in structure fires, including the sensing principles, fabrication, key characteristics, and recently-reported applications. Three categories of fiber optic sensors are reviewed: Grating-based sensors, interferometer sensors, and distributed sensors.

## 1. Introduction

Fire can cause structural damage with catastrophic consequences. The strength and stability of structures can be significantly impacted by the adverse effects of temperature-induced deformations and degraded material properties at elevated temperatures [1,2,3,4]. In a structure fire, room gas temperatures from 600 °C to 1200 °C are common [5]. Concrete begins to lose significant strength when the material temperature exceeds 350 °C and ceases to be structurally useful for temperatures more than 550 °C [6]. Structural steels are commonly considered to have lost half of their room temperature yield strength around 540 °C [7] and have lost the majority of their strength above 700 °C. To assess the thermo-mechanical conditions of a structure during or after a fire, temperatures and strains are critical [8,9]. However, the extreme environmental and material temperatures, as well as large transient temperature variations present in a fire, make monitoring of temperatures and strains challenging. 

Traditional measurement tools for structural fire engineering include thermocouples for temperature measurement, strain gauges for local deformation measurement, and displacement transducers for the measurement of global displacements [10]. These sensors, which utilize electrical signals, typically have a limited operating temperature range and can be susceptible to electromagnetic interference. Furthermore, they provide measurements only at a single point. Because the properties in a structure often vary across the physical scale of interest, multiple data points are necessary to adequately characterize the property. Consequently, a great number of sensors must be deployed to obtain measurements at multiple locations; which involves cumbersome wiring of the sensors in large specimens. 

In recent years, fiber optic sensors have drawn interest for the monitoring of structures in fire environments due to their unique characteristics such as immunity to electromagnetic interference (EMI), compact size, and stability in harsh environments [11]. These advantages motivate this review, which aims to inspire further advancement of fiber optic sensor technology and to provide guidance for the selection and use of fiber optic sensors for structural fire applications.

The paper provides a brief introduction intended for structural fire engineers of the basic concepts of fiber optic sensors and then describes the three primary types of fiber optic sensors that have been applied in structural fire applications in more detail. For each sensor type, the paper reviews the sensing principle, fabrication, key characteristics, and recent structural fire applications. In addition to the fiber optic sensors that have been applied in structural fire engineering, some sensors that are promising for this application, but have not been applied in fires, are also discussed. While interesting studies on sapphire optical fibers have been conducted in recent years [12,13], application of sapphire-based optical fibers in structural fire engineering remains rare. This review focuses on fiber optic sensors made of fused silica fibers. 

## 2. Basic Concepts of Fiber Optic Sensors

Fiber optic sensors function based on measuring the characteristics of light transmitted along an optical fiber. Changes in the fiber caused by external actions, for example, changes in the temperature or stress state of the fiber, resulting in variations in the transmitted light signal that can be calibrated to the measurand. Different fiber optic sensor types utilize different physical phenomena to modulate the transmitted light; which gives each sensor its characteristic performance. Relevant functional principles for this review are discussed in the following sections. For a general discussion of fiber optic sensors, a wealth of literature is available [14,15,16,17]. 

**Figure 1:**
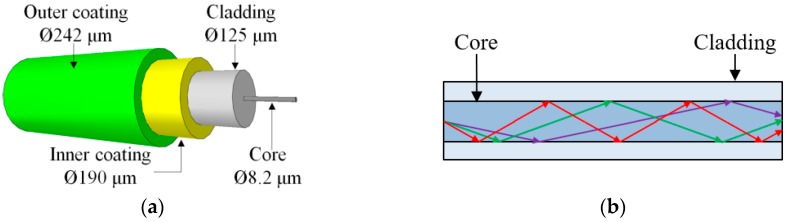
Optical fiber: (**a**) Typical cross section; (**b**) waveguide principle.

Optical fibers can be categorized into single-mode fibers (SMFs) and multi-mode fibers (MMFs) according to the number of waveguide modes in the fiber. Typically, SMFs consists of a fiber core (8 μm to 10 μm in diameter), a cladding (≈125 μm in diameter), and one or more layers of protective coating, as illustrated in Figure 1a. For fused silica fibers, the fiber core and cladding are made of high-purity fused silica, doped with germanium to increase the refractive index; although other dopants may be used. Light waves are transmitted along the fiber through total internal reflection at the core-cladding interface as illustrated in Figure 1b. Multi-mode fibers have a larger diameter core that allows multiple propagation modes of the guided wave (Figure 1b). Compared with MMFs, SMFs have higher transmission speed and lower transmission loss, and thus generally more suitable for long-distance applications.

Three categories of fiber optic sensors have been reported in the context of structural fire engineering: Grating sensors, interferometer sensors, and distributed sensors [18]. Grating sensors and interferometer sensors are referred to as “point sensors” because they only measure at the locations where a grating or interface is present. Some point sensors can be multiplexed to form a quasi-distributed sensor for measurements at multiple locations [19]. Distributed fiber optic sensors provide measurements at multiple points along the optical fiber.

## 3. Grating Sensors

Optical fiber gratings are made by periodic perturbation of the refractive index along a length of optical fiber. The phase-matching condition of gratings can be described by Equation (1) [20]:

(1) $$\beta_{1}-\beta_{2}=\Delta \beta =2\pi /\Lambda$$

where Λ represents the period of the grating; β_1_ and β_2_ are the propagation constants of the coupled modes, and Δβ is the difference between the two propagation constants. Depending on the period length, fiber gratings can be classified into fiber Bragg gratings (FBGs) and long-period fiber gratings (LPFGs). In an FBG, the forward-propagating fundamental mode (LP_01_, β_1_ = β_01_) couples to the reverse-propagating fundamental mode (β_2_ = −β_01_ < 0), resulting in a large Δβ and a period that is typically less than 1 μm. LPFG couples the fundamental mode to forward-propagating cladding modes (β_2_ > 0), resulting in periods that are typically greater than 100 μm. The longer period leads to a longer grating length, which results in a longer sensor length and less precise sensing location. 

### 3.1. Fiber Bragg Grating Sensors

#### 3.1.1. Principle

**Figure 2:**
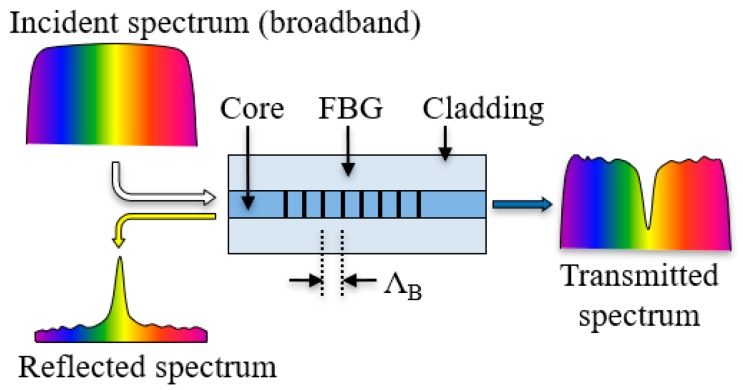
Illustration of reflection of fiber Bragg gratings (FBG).

In an FBG sensor, a narrow band of the incident optical field within the fiber is reflected by successive, coherent scattering from the index variations, as illustrated in Figure 2. The strongest interaction, or mode coupling, occurs at the Bragg wavelength [21], as described by Equation (2):

(2) $$\lambda_{B}=2n_{eff}\Lambda_{B}$$

where λ*_B_*, *n_eff_*, and Λ*_B_* represent the Bragg wavelength, the effective refractive index of the fiber core, and the period of the grating, respectively. 

The effective refractive index of the fiber core and the period of grating, *n_eff_* and Λ*_B_*, vary with the fiber temperature and strain. In a structural fire testing, the temperature and strain of the sensor can be determined by measuring the central wavelength shift of the FBG, provided the temperature and strain sensitivity coefficients that are calibrated before the testing. The effects of temperature and strain on the fiber can be decoupled by measuring the temperature separately using a second sensor for temperature compensation.

#### 3.1.2. Fabrication and Key Characteristics

FBGs are typically about 5 mm in length and fabricated using a phase-mask approach to obtain gratings with controlled spectral response [22]. To generate phase-mask gratings, a laser is focused on the fiber core through a phase mask that causes an interference pattern and inscribes multiple points. This requires the fiber core to be photosensitive. Another method to fabricate gratings is the point-by-point writing approach; where the fiber core is inscribed one point at a time using a laser. Point-by-point writing is inefficient for gratings with many index perturbations, such as FBGs; however, it is often appropriate for making LPFGs. 

Traditionally, FBGs have been divided into two categories based on the formation mechanisms: Type I and Type II [23]. Conventional Type I gratings are formed through a single ultraviolet (UV) photon absorption process that excites oxygen deficiency centers in the fiber core [24]. At elevated temperatures, Type I gratings degrade as a result of the thermal depopulation of trapped excited states that are created during the grating formation, because atoms at excited state can absorb energy and return to the ground state. Therefore, Type I gratings are typically not applicable for measurements over 400 °C. The refractive index change of Type I FBGs is almost completely annealed at temperatures over 600 °C. Type II gratings use high-power pulsed UV laser sources to locally damage the fiber core or the core-cladding interface for the formation of periodic perturbation on refractive index [25]. Type II gratings in fused silica fiber have been used for temperature measurements higher than 1000 °C [26]. Therefore, the fabrication technique significantly influences the performance of the gratings and must be considered when selecting FBG sensors for structural fire applications. Furthermore, the stability and uncertainty of the measure must be considered for the range of application conditions. 

Regenerated FBGs (RFBGs) fabricated through a hydrogen loading process are reported in [27,28,29]. A hydrogen loading process requires an optical fiber to be exposed to high pressure hydrogen gas at room temperature until saturated with hydrogen that permeates the glass matrix. The fiber is then exposed to UV irradiation that results in the formation of highly reflective seed gratings, which are then completely or partially removed in a thermal annealing process [23]. Further heating at high temperature regenerates gratings, which have smooth spectra and are stable at temperatures above 1000 °C [30,31]. 

#### 3.1.3. Structural Fire Applications

**Figure 3:**
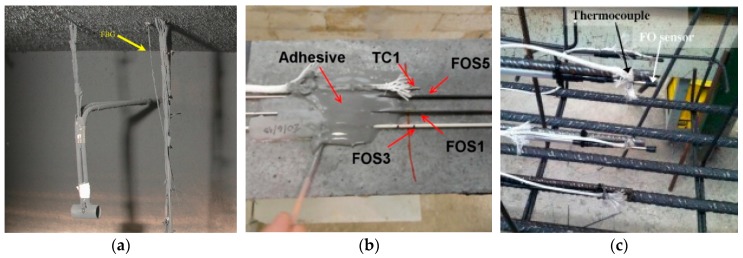
Application of FBG sensors in structural fire testing: (**a**) Gas temperature [11], (**b**) surface-attached on concrete beams [35], and (**c**) embedded in reinforced concrete beams [36].

FBG sensors have been applied to measure gas temperature in a scaled (1:20) tunnel [11], and the measurements were compared with measurements from type-K thermocouples and theoretical (predicted) temperatures. The maximum operating temperature of the FBG sensors was 350 °C. The FBG sensors were attached to a bundle of wires suspended in the tunnel, with no additional protection, as shown in Figure 3a. During the fire test, the FBG sensors measured temperatures up to 300 °C. Compared with the theoretical temperatures, the authors show that the results from the FBG sensors were more accurate than the measurement from the uncorrected thermocouples, which were subjected to radiation-induced temperature reduction. In the study [11], potential strain effect on the measurement from the FBG sensors was not considered, although the ventilation-induced air flow might impact the strain and thus center wavelength in the FBG sensors.

As discussed in the introduction, gas temperatures higher than 350 °C can be expected in a fire environment. With a regenerated FBG, temperature measurements up to 1295 °C were demonstrated using a microheater [32]. Bueno et al. [33] tested three types of FBGs embedded in concrete cylinders under fire. The three types were (1) FBG inscribed in photosensitive germanium-boron co-doped fiber, (2) regenerated FBG inscribed in germanium doped fiber, and (3) regenerated FBG in germanium-boron co-doped fiber. The FBG sensors were embedded in the concrete cylinders without additional protection and a survival rate of 92%. The concrete cylinders were placed in a furnace where the temperature was increased above 1000 °C, following the standard ISO 834 fire curve [34]. The testing was continued for 120 min. The temperatures measured from the FBGs were compared with the temperatures measured from type-K thermocouples. While the ordinary FBGs were annealed (and ceased to measure) at 420 °C, the measurement from the regenerated FBGs agreed with the measurement from thermocouples above 1000 °C, demonstrating the viability of the regenerated FBGs at these temperatures. 

Following the above study, the same research group conducted more fire testing of concrete beams using the regenerated FBG [35,36]. Rinaudo et al. [35] installed regenerated FBG sensors, which were packaged for mechanical and fire protection, on the surface of concrete beams using a metallic composite adhesive (Figure 3b), which retains an adequate bond with concrete at up to 1093 °C. The authors claimed that the regenerated FBGs measured the gas temperature, although the FBGs were encased in the package. Type-K thermocouples were closely installed on the concrete beams to measure gas temperature for comparison. Two concrete beams were tested in a furnace for one hour, following the standard ISO 834 fire curve [34]. The regenerated FBGs measured temperatures up to 990 °C and showed agreement with the measurement from the type-K thermocouples. Torres Górriz et al. [36] embedded the packaged regenerated FBGs in a reinforced concrete beam subjected to elevated temperatures, as shown in Figure 3c. To protect the sensors and transmission cable, they were passed through a flexible PVC tube, which was attached to the steel bars for mechanical protection during concrete casting and might decompose as the temperature exceeded 120 °C. Type-K thermocouples were used to measure temperature for comparison. The beam was tested in a fire for 77 min, following the standard ISO 834 fire curve. The temperature measured by the regenerated FBGs was up to 953 °C, and overall in agreement with the measurement from the thermocouples. The authors learned that the transmission cable of the fiber optic sensor should be carefully protected throughout the sensor installation, specimen fabrication, and fire testing. 

Advantages of FGB sensors for structural fire applications include: (1) Lower cost electronics (measurement system) compared to other fiber optic technologies, (2) good sensor reliability, and robustness due to the high sensitivity to temperature and insensitivity to other environmental variables such as refractive index, and (3) ease of sensor packaging and installation due to the short sensor length. A primary drawback is that FGB gratings are annealed (erased) at temperatures too low for most applications in the fire. While this drawback can be overcome using regenerated gratings, the fabrication process is cumbersome and the resulting gratings have low reflectivity, which limits their ability to be multiplexed. 

#### 3.1.4. Other Promising FBG Technologies

Micro-fabrication techniques using femtosecond lasers have been exploited to inscribe FBGs in optical fibers without the requirement of photosensitivity of the fiber core [37,38]. Both Type I and Type II gratings have been inscribed using femtosecond lasers and a phase mask [39]. During the microfabrication process, an optical fiber absorbs energy from the femtosecond laser through nonlinear phenomena such as multiphoton, tunneling, and avalanche ionization. The absorbed energy locally damages the fiber, which leads to the formation of void-like grating structures [38]. FBGs fabricated in fused silica fibers using femtosecond lasers can provide stable temperature or strain measurements at an elevated temperature of 1000 °C for about 400 h, but a permanent drift of the central wavelength was observed at 1050 °C [40]. Thermal annealing at high temperature has been used to enhance the thermal stability of FBGs fabricated using femtosecond lasers [41]. After pre-annealing at 1100 °C, the FBGs demonstrated sustained reflectivity up to 1200 °C for more than 20 h [41]. Despite the great potential, the microfabricated FBGs have not been applied in structural fire testing, likely due to the fabrication cost, thus their feasibility must be further verified.

The upper operating temperature of FBGs is limited by the softening point and thermal degradation of the fiber material, such as de-vitrification. The softening point of silica fibers is between 1200 °C and 1600 °C depending on the type and content of additive materials. Single crystal sapphire has a melting point around 2050 °C and thus might possibly increase the temperature limit. FBGs inscribed in sapphire fibers have been reported for measurements up to 1850 °C [12,13,14]. Femtosecond lasers were used to fabricate the FBGs in sapphire fibers since they provided high peak intensities and multi-photon processes to permanently change the refractive index. However, sapphire fibers do not have fiber cladding and are multimode fibers, which involve more significant signal loss and require more complex interrogators. Light waves are constrained by the interface between the fiber core and the environment, and thus, the light waves are sensitive to the environmental conditions and surface defects. Such sensitivity must be considered in structural fire applications, since other environmental variables and surface conditions may affect the measurement. 

### 3.2. Long Period Fiber Grating Sensors

#### 3.2.1. Principle, Fabrication, and Key Characteristics

There are two waveguide interfaces in an optical fiber with a long-period fiber grating (LPFG): (1) The core surrounded by a cladding, and (2) the cladding surrounded by the environment (e.g., air). Phase matching of the guided core mode and a forward-propagating cladding mode of an LPFG occurs at the resonance wavelength λ, which is described in Equation (3) [42]:

(3) $$\lambda =\left[n_{eff}\left(\lambda \right)-n_{clad}^{i}\left(\lambda \right)\right]\Lambda$$

where *n_eff_*(λ) is the effective refractive index of the propagating core mode at the wavelength λ, *n^i^_clad_*(λ) is the refractive index of the i^th^ cladding mode, and Λ is the period of the grating.

The high attenuation of the LPFG cladding modes results in a series of attenuation bands in the transmission spectrum of the fiber at discrete resonant wavelengths; each attenuation band corresponds to the coupling to a different cladding mode [42]. The resonant wavelengths of the attenuation bands are sensitive to the period of the LPFG and the effective refractive index with influences from the local environment: Temperature, bending radius, and the refractive index of the surrounding medium [43]. Typically, a higher order of coupling mode corresponds to a greater resonant wavelength and larger intensity of transmission loss. As temperature or strain changes, the resonant wavelengths are shifted, and the shifted quantity of resonant wavelength can be measured to calibrate the temperature and strain sensitivity of the LPFGs.

The period of LPFGs is in the range from 100 µm to 1 mm, and the length is in the order of 30 mm. Similar to FBG, the phase mask technique and point-by-point writing technique can be used to fabricate LPFGs [42]. While the phase mask and UV exposure method are widely used for fabricating FBGs, the point-by-point writing technique is more convenient to use for LPFG fabrication because of the relatively large periodicity. In addition to UV irradiation, CO_2_ laser [44], femtosecond laser [45], and electric arc fabrication [46] have also been used to fabricate gratings. In a photonic crystal fiber, LPFGs were fabricated by periodically collapsing the holes of the fiber using a CO_2_ laser [47]. LPFGs fabricated by CO_2_ laser irradiation are reported to be stable up to 1200 °C [44]. Femtosecond lasers were used to fabricate LPFGs based on densification of the glass, and the LPFG allowed for measurements at temperatures up to 500 °C [45]. Electric arc fabrication of an LPFG relies upon a combination of several different mechanisms to generate the periodic property modulation of the fiber. The mechanisms include the induction of microbend into the fiber [48], the periodic tapering of the fiber [49], the diffusion of dopants [50], ion implantation [51], and the relaxation of internal stresses [52]. The operating temperature range of such LPFGs has been demonstrated to be up to 800 °C without permanent modification of their properties [53], and, if annealed they may operate at temperatures up to 1190 °C [46].

#### 3.2.2. Structural Fire Applications

Recently, an LPFG sensor was inscribed in a fused silica SMF using a CO_2_ laser and deployed for high temperature and large strain measurement [54]. Although the fused silica fiber with LPFGs is fragile and prone to fracture under mechanical loading, an innovative mechanism for scaling down the thermal elongation was designed to delay the strain increase and protect the LPFG from a fracture at high temperature. The LPFG sensors inscribed using the CO_2_ laser were operated at temperatures up to 700 °C. 

**Figure 4:**
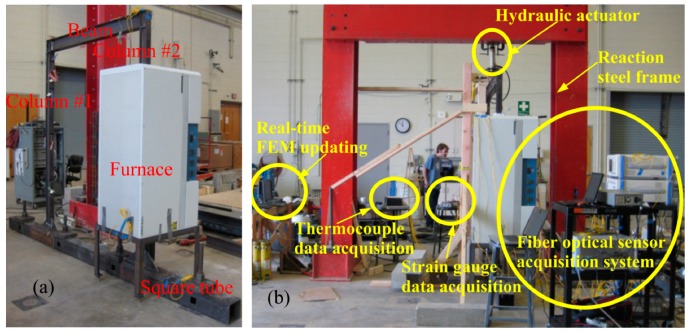
Application of long-period fiber gratings (LPFG) sensors in structural fire [56]: (**a**) Test set-up, and (**b**) instrumentation.

Following the same concept, the LPFG sensor was improved and used to measure temperatures up to 800 °C, as elaborated in reference [55]. Then, the LPFG sensors were applied in a large-scale steel frame for temperature measurement in a simulated fire environment (Figure 4). One of the columns was partially exposed to the fire effect to investigate the thermomechanical behavior of a steel frame under the fire [56]. A two-part ceramic adhesive that can endure temperatures up to 1100 °C was used to attach the LPFG sensors to the inside surfaces of the column flanges. The sensor data were used for real-time updating of a finite element model to accurately predict the thermomechanical responses of the steel frame under a fire. The LPFG sensors measured temperatures up to 800 °C. 

Compared with FBG sensors, the application of LPFG sensors in structural fire research is relatively new. Although the fabrication of LPFGs is straightforward and cost-effective, LPFGs are sensitive to bending of the optical fiber and refractive index of the environment, implying that more attention must be paid to the sensor installation and data interpretation. For a successful structural fire application, the LPFGs must be properly packaged to minimize undesired bending and environmental influences on the refractive index and to enhance the mechanical strength and ruggedness of the sensor. However, the sensitivity to bending and refractive index offers opportunities to develop multifunctional LPFG sensors, for example, to measure refractive index, rotation due to bending, and temperature simultaneously, although the feasibility remains unclear. 

## 4. In-line Interferometer Sensors

A fiber optic interferometer uses the interference between two coherent light beams traveling along different paths to measure the variables that alter the interference pattern. Four types of fiber optic interferometers have been demonstrated: Fabry-Perot interferometers (FPIs), Mach-Zehnder interferometers (MZIs), Michelson interferometers (MIs), and Sagnac interferometers (SIs). An FPI is formed by light reflected from two parallel surfaces at discontinuities along the beam paths. For MZIs, MIs, and SIs, the interference is normally generated by splitting an incident light into two paths using a fiber splitter followed by recombining them using a fiber combiner. Early MZIs, MIs, and SIs had two arms, and thus had disadvantages such as complicated structure, large size, and high susceptibility to environmental changes. Recently, various in-line fiber optic core-cladding-mode interferometers (CCMI) have been developed to replace the two-arm structures. The CCMI operates on the interference between the core and the cladding modes in a single optical fiber. In structural fire applications, different interferometer sensors are suited to measuring temperature or strains.

### 4.1. In-line Fabry-Perot Interferometer Sensors

#### 4.1.1. Principle

**Figure 5:**
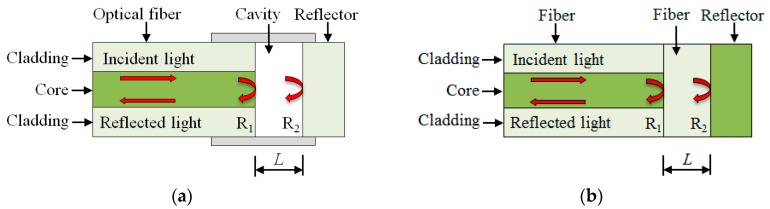
Illustration of Fabry-Perot Interferometers (FPIs): (**a**) Extrinsic (E)FPI; (**b**) intrinsic (I)FPI.

Interference occurs when a reflected light and a transmitted light are coherent at two reflecting surfaces (R1 and R2 in Figure 5). The reflection spectrum of an FPI can be described as the wavelength dependent intensity modulation of the input light spectrum [57], which is mainly caused by the optical phase difference between two reflected light beams. Constructive interference occurs if the reflected beams are in phase, and this corresponds to a high-reflection peak. If the reflected beams are out-of-phase, destructive interference occurs and this corresponds to a reflection minimum. Whether the reflected beams are in phase depends on the wavelength (λ) of the incident light (in vacuum), the angle (θ) with which the incident light travels through the reflecting surfaces, the length (*L*) between the reflecting surfaces, and the refractive index (*n*) of the material between the reflecting surfaces. The phase difference between each reflected pair of an FPI is given as [58]:

(4) $$\phi =\frac{2\pi }{\lambda }2nL\cos(\theta )$$

When any perturbation is introduced to an FPI, the phase difference is influenced by the variation in the optical path distance of the interferometer [59]. For example, applying a longitudinal strain to an FPI sensor changes *L*, which in turn results in phase variation. By measuring the shift of the wavelength, the applied strain can be quantified. 

A number of techniques have been reported to form FPIs, such as thin film deposition [60,61], forming a micro-notch using femtosecond lasers [62], offset structures [63], chemical etching [64,65], and splicing [66]. Depending on the physical structure of an FPI, in-line FPIs can be categorized into extrinsic FPI (EFPI) and intrinsic FPI (IFPI). As illustrated in Figure 5, an EFPI has a cavity between the reflecting surfaces (R1 and R2), while an IFPI does not have the cavity.

#### 4.1.2. Structural Fire Applications and Key Characteristics

**Figure 6:**
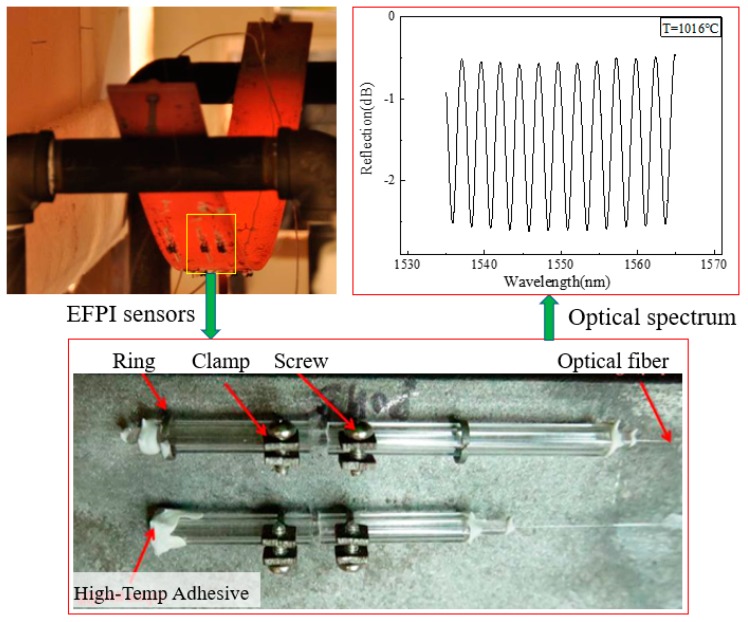
Application of EFPI for large strain measurement in a fire experiment of steel plates.

Due to the discontinuity of the optical fiber at the cavity, the physical strain limitation of the optical fiber, and thus, EFPIs can be used to measure large strains. The reflector can be a cleaved end of an optical fiber or a mirror with a high reflectivity (Figure 5a). The use of a mirror helps minimize the size of the sensor, facilitating sensor installation in structures. Customized EFPIs have been used to measure the strain of steel plates in a fire [67], as shown in Figure 6. The steel plate measured 10 mm in thickness and was simply supported on two steel pipes with a span length of 1.42 m. According to temperature sensors in the testing [67], both the gas temperature and the temperature in the steel plate were above 1000 °C. Under gravity, creeping occurred at elevated temperatures and the steel plate exhibited a large deformation (Figure 6). The EFPI sensors were installed on the surface of the steel plate through a mechanical mechanism. The EFPI sensors were assembled using silica tubes and a two-part ceramic adhesive and installed using steel clamps and rings that were fixed on the steel plate with drilled holes. The EFPI sensors measured the reflection spectrum at temperatures above 1000 °C, and the spectrum was used to determine the strain change in the steel plate. 

EFPI sensors have also been used to measure strain and crack opening in ceramic materials subjected to coal fire [68]. Crack opening widths in a range of 17 μm to 33 μm were measured using EFPI sensors at temperatures up to 900 °C. EFPI sensors with a similar scheme have been used to measure strains of steel frames [56]. The sensors attached on the surface of the steel columns measure large strains up to 10% at temperatures up to 800 °C. 

Primary disadvantages of EFPIs include low coupling efficiency, and they require careful alignment of the reflectors. The installation of these sensors can be slow and challenging, and attention must be paid to the sensor packaging [59]. Another problem for EFPI sensors with un-sealed cavities learned from live fire experiments, is that exposed reflecting surfaces can become contaminated by soot. 

For IFPI, the sensing element is a short fiber sandwiched between two reflecting surfaces. The light waves of an IFPI propagate in the fiber core only, achieving higher signal intensities with a better signal demodulation compared with the EFPIs. An IFPI sensor was fabricated by creating an internal mirror in the fiber by depositing a thin layer (≈100 nm) of TiO_2_, which had an operation temperature up to 1050 °C [60]. Following the same concept, an IFPI with a thin layer of Cr operated at 1100 °C with a stability of 10 °C for a duration of more than 300 h [61]. Micro-machining using a femtosecond laser was used to fabricate miniature IFPIs operated at temperatures up to 1100 °C [62]. An easy-to-fabricate IFPI has been presented by splicing two sections of two fibers with a large lateral offset (≈62.5 μm) and allows for strain measurements at temperatures up to 1000 °C [63]. Recently, photonic crystal fibers (PCFs) have attracted interest because of their unique waveguide mechanisms and modal properties. A section of PCF can be spliced to a single mode fiber to form an IFPI that can operate at temperatures up to 1200 °C [69]. Miniature IFPIs with a micro air bubble can be formed by splicing PCF to a single mode fiber [70,71,72] to work at temperatures up to 1000 °C [73]. Sapphire fibers have been used to fabricate IFPIs that can measure temperatures exceeding 1000 °C [74,75]. 

Despite the above technology advancement, IFPIs have not been applied to structural fire engineering. Compared with the EFPIs, an IFPI typically requires expensive equipment, special fibers, or toxic chemicals to fabricate the reflecting surfaces. Another reason is that the IFPIs are relatively new, and need further research to standardize the fabrication procedure and verify the performance in structural fire applications. Finally, the optical fiber of an IFPI must be continuous, which limits the strain measurement capability. However, compared with grating sensors, IFPIs have higher thermal stability, because the cavities generally are more thermally stable than the gratings, although the regenerated FBG may be very thermally stable, as indicated in reference [32]. 

### 4.2. In-Line Fiber Optic Core-Cladding-Mode Interferometers

#### 4.2.1. Principle

**Figure 7:**
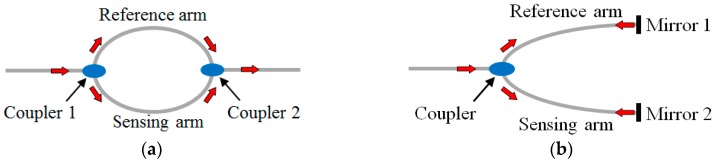
Illustration of Core-Cladding-Mode Interferometers (CCMIs): (**a**) Mach-Zehnder interferometer; (**b**) Michelson interferometer.

In-line CCMIs require a splitting and re-coupling mechanism between the core and cladding modes, which are guided by the core-cladding and cladding-ambient interfaces, respectively. The differential phase of the core and cladding modes allows CCMIs to sense numerous environmental parameters. CCMI sensors have been demonstrated to form MZIs and MIs, as illustrated in Figure 7a,b, respectively. Both structures involve a reference arm and a sensing arm. The main difference between MZIs and MIs is that an MZI requires two couplers, while an MI only needs one coupler that splits and re-combines the two beams due to the use of mirrors in the reference and sensing arms. MZI and MI sensors measure the transmitted signals and reflected signals, respectively.

In an MZI, a beam splitter partially couples the core mode to the cladding modes and a combiner recombines the cladding modes to the core mode. The phase difference of the core and cladding modes can be described as:

(5) $$\Phi_{MZI}=\frac{2\pi \left[n_{eff}\left(\lambda \right)-n_{clad}^{i}\left(\lambda \right)\right]L}{\lambda }$$

where *n_eff_*(λ) is the effective refractive index of the propagating core mode, *n^i^_clad_*(λ) is the refractive index of the i^th^ cladding mode, and *L* is the fiber length between the splitter and the combiner.

In an MI, each beam is reflected at the end of each fiber arm. The phase difference of the core and cladding modes can be described as:

(6) $$\Phi_{MI}=\frac{4\pi \left[n_{eff}\left(\lambda \right)-n_{clad}^{i}\left(\lambda \right)\right]L}{\lambda }$$

#### 4.2.2. Fabrication

**Figure 8:**
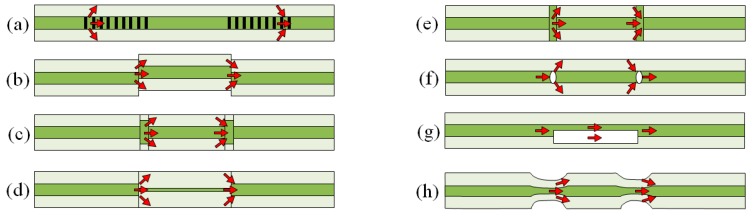
Configurations for in-line Mach-Zehnder interferometers: (**a**) A pair of LPFGs; (**b**) core-offset; (**c**) core diameter mismatch with larger core fiber; (**d**) core diameter mismatch with smaller core fiber; (**e**) air-hole collapsing of PCF; (**f**) cavities formed in core by femtosecond laser; (**g**) cavity formed at core-cladding interface by femtosecond laser; (**h**) fiber tapering.

Based on the principle, different innovative strategies have been presented to realize the CCMIs. A pair of LPFGs can be used as the splitters/combiners to form an MZI, as illustrated in Figure 8a [76]. The operating temperature of the MZI is therefore limited by the temperature stability and other limitations of LPFGs aforementioned. Another method to couple the core mode to cladding modes is to splice two fibers with a small lateral offset, as depicted in Figure 8b. The number of coupled cladding modes can be controlled by adjusting the amount of offset. Using fibers with different core sizes is an alternative to split the beam in a fiber as shown in Figure 8c,d. In Figure 8c, a short multimode fiber is spliced to a conventional SMF at two points [77,78]. In Figure 8d, a length of fiber with a smaller core is spliced in between two conventional single mode fibers [79]. Collapsing air holes of a PCF is another way of making an in-line MZI, as shown in Figure 8e [80]. Cavities in the fiber can also be formed using a femtosecond laser as seen in Figure 8f,g to create the splitters/combiner pairs [81,82]. Finally, tapering of the fiber can also be used to create an MZI as shown in Figure 8h [83,84]. The tapering increases the core mode diameter. 

**Figure 9:**
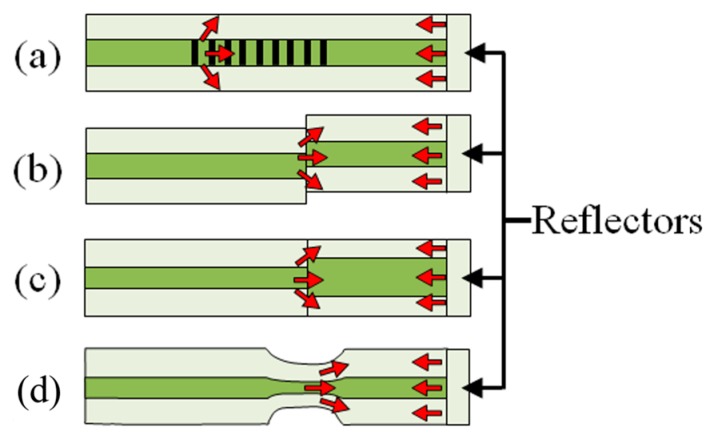
Example configurations for in-line Michelson interferometers: (**a**) Long-period fiber grating; (**b**) core-offset; (**c**) core diameter mismatch; (**d**) fiber tapering.

The structure of an MI works as half of an MZI, as illustrated in Figure 9a,c [85,86]. The fabrication methods and the operation principle of MIs are similar to MZIs. Since MIs use reflection modes, they are more compact than MZIs. A fiber taper MI sensor was fabricated in a single more fiber by a fiber-taper machine and electric-arc discharge [86,87,88]. 

#### 4.2.3. Structural Fire Applications

Except for the designs involving LPFGs (Figure 8a and Figure 9a), which depend on the thermal degradation of the LPFGs, the CCMIs reviewed have potential operation temperatures greater than 1000 °C. For instance, research has demonstrated that the core mismatch scheme (Figure 8b and Figure 9b) does not degrade until the intrinsic degradation of the optical fiber [79,81,82,89]. The MZI formed by splicing a smaller core is spliced in between two conventional single mode fibers operated at temperatures up 1000 °C [79]. The MZIs based on cavities fabricated using a femtosecond laser operated at temperatures up to 1200 °C [81,82]. The fiber taper MI sensor fabricated by a fiber-taper machine and electric-arc discharge was used to measure the temperature at up to 1000 °C [89].

To the author’s knowledge, CCMI sensors have not been used in structural fire applications. While many innovative schemes have been proposed to realize the CCMIs, further research is needed to understand their performance in fires. 

## 5. Distributed Fiber Optic Sensors

In addition to transmitting signals from point sensors (e.g., grating sensors and interferometers), optical fibers can be used as distributed fiber optic sensors based on light scatterings [90], i.e., with no inscribed grating or reflection surface. The absence of gratings or reflection surfaces makes this approach attractive for high-temperature applications. Moreover, thousands of sensing points can be achieved in a single fiber. Distributed fiber optic sensors have attracted interest outside of the field of structural fire research due to the low cost of the sensor (a telecommunication-grade single-mode fiber that costs a few cents per meter serves as a sensor) and its fully-distributed sensing ability. However, the data analyzers are currently relatively expensive compared to grating sensor systems. 

**Figure 10:**
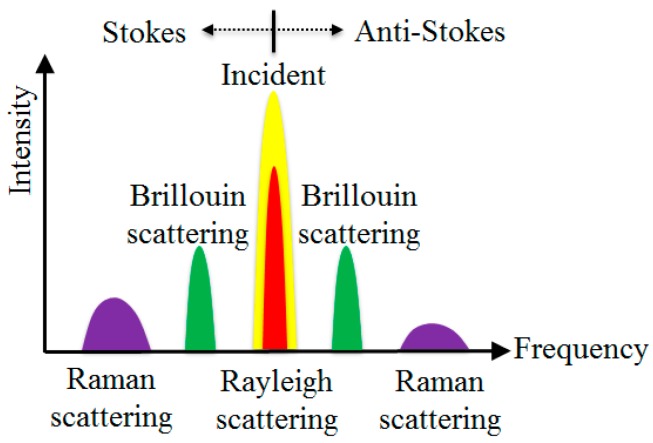
Light scatterings in optical fiber.

When light propagates through an optical fiber, it interacts with the atoms (or molecules) in the fiber, generating scattering signals that can be used to perform sensing [18]. There are three types of scattering: Rayleigh, Raman, and Brillouin scatterings, as illustrated in Figure 10. Rayleigh scattering results from the interaction between light waves and tiny particles inherent in the fiber. It is an elastic scattering process that does not involve a frequency change. Unlike Rayleigh scattering, Raman and Brillouin scattering involve frequency changes. Brillouin scattering is obtained by the interactions between light waves and acoustic waves, and Raman scattering is introduced by the interactions between light waves and molecular vibrations. Various distributed fiber optic sensing technologies have been developed based on these scattering phenomena [18]. When assessing the technologies for a particular application, one must consider tradeoffs between accuracy, spatial resolution, temporal resolution, and system cost.

### 5.1. Rayleigh Scattering Based Sensing Technologies

The intensity of Rayleigh backscattering is mapped along the length of an optical fiber using optical time domain reflectometry (OTDR). Coherent OTDR has been developed by mixing the backscattered and reference light with a coherent detection technique [91]. Because the distances between scattering centers (i.e., inclusions in the fiber) are smaller than the wavelength of light, the secondary light waves from Rayleigh scattering are coherent. Rayleigh OTDR has been applied to measure temperature, strain, and displacement [92,93,94,95]. The spatial resolution is related to the signal pulse width and thus the bandwidth of the detector, electrical amplifier, and digitizer. Millimeter-scale spatial resolution, which is often required for structural applications, requires a bandwidth in the order of tens of GHz, which necessitates a sophisticated (and often expensive) acquisition system [18]. Alternatively, optical frequency domain reflectometry (OFDR) converts the frequency response to the time domain using Fourier transforms thus that the spatial resolution with OFDR does not depend on the bandwidth of the detector or digitizer [94,96]. OFDR has been applied to measure temperature and strain with a centimeter-scale spatial resolution [97], and the measurement distance is typically up to hundreds of meters. 

### 5.2. Raman Scattering Based Sensing Technologies

The intensity of Raman scattering is dependent on the fiber temperature, which provides the physical basis for the measurement of absolute temperature [98]. Based on Raman scattering, Raman optical time domain reflectometry (ROTDR) and Raman frequency time domain reflectometry (ROFDR) have been developed for temperature measurement. Due to the weak scattering intensity, the spatial resolution of ROTDR is typically limited to about 1 m with a measurement distance of 10 km. Recently, a superconducting nanowire single-photon detector was developed and utilized to improve the spatial resolution of ROTDR to the order of 1 cm at 1550 nm wavelength [99]. This development potentially can facilitate wider applications of Raman scattering based distributed sensors for structural fire applications in the future; however, sampling times need to be significantly reduced to make this technology applicable in structural fire applications.

### 5.3. Brillouin Scattering Based Sensing Technologies

The distribution of Brillouin scattering spectrum was first measured through Brillouin optical time domain analysis (BOTDA) [100]. BOTDA measures backscattering signals in an optical fiber due to the combined effect of strain and temperature as a result of a forward-propagating (incipient) pulse pump light wave and a back-propagating continuous probe wave [18]. If the pump and probe waves are tuned to have time-varying frequency differences corresponding to the Brillouin frequency of optical fiber, the Brillouin gain as a function of position can be determined by the time-varying probe wave. The Brillouin frequency can be mapped along the optical fiber, which enables measurement of strain and temperature distributions [101,102]. Brillouin optical time domain reflectometry (BOTDR) has the advantage of single-ended measurement [103]. However, the spatial resolution is on the order of meters, which is mainly limited by the pulse width. While a narrow bandwidth pulse can lead to higher resolution, the bandwidth must be longer than the phonon relaxation time. It has been demonstrated that 28 ns is required to get the phonon fully stimulated, which corresponds to a 3 m spatial resolution. To resolve this problem, pulse pre-pump BOTDA (PPP-BOTDA) was proposed [104], which uses a pre-pump pulse that stimulates the phonon before a narrow bandwidth pulse arrives, achieving a 2 cm spatial resolution using a 0.2 ns short pulse. On the other hand, frequency domain distributed sensing technologies have been developed such as Brillouin optical frequency domain analysis (BOFDA) [105] and Brillouin optical correlation domain analysis (BOCDA) [106]. In a short distance (e.g., ≈10 m), the spatial resolution can achieve 3 cm by BOFDA and 1 cm by BOCDA [107]. However, the spatial resolution can be up to 7 cm for a 1 km measurement distance [108]. 

### 5.4. Structural Fire Applications

The capability of measuring temperature distributions along the whole fiber length makes distributed fiber optic sensors well-suited for fire detection in structures such as tunnels [109,110,111,112,113,114]. Rayleigh OFDR was used to monitor temperature distribution for a nuclear reactor with a spatial resolution of 1 cm up to 850 °C [97]. ROTDR was operated at temperatures up to 1000 °C with a temperature uncertainty of less than 30 °C [115]. BOTDA was investigated for measuring temperature distributions in an electric furnace at up to 1000 °C using a fused silica single mode fiber [116,117,118,119,120]. In addition to measuring temperature, Bao and Chen investigated the strain sensing performance of a distributed fiber optic sensor at temperatures up to 800 °C, and temperature dependency of the strain and temperature sensitivity coefficients was quantified using PPP-BOTDA [121]. Brillouin frequency was measured and converted into strain and temperature distributions along the optical fiber with temperature compensation [122,123,124]. Temperature compensation can be realized using an additional distributed fiber optic sensor free of strain effect for temperature measurement or using two different distributed sensing approaches with different temperature and strain sensitivities [18]. For example, Rayleigh OFDR and BOTDA have different temperature and strain sensitivities, thus temperature and strain can be solved for each point. The distributed fiber optic sensor was applied to measure the coefficient of thermal expansion of steel [121]. These studies demonstrated the feasibility of distributed fiber optic sensors for high temperature applications.

**Figure 11:**
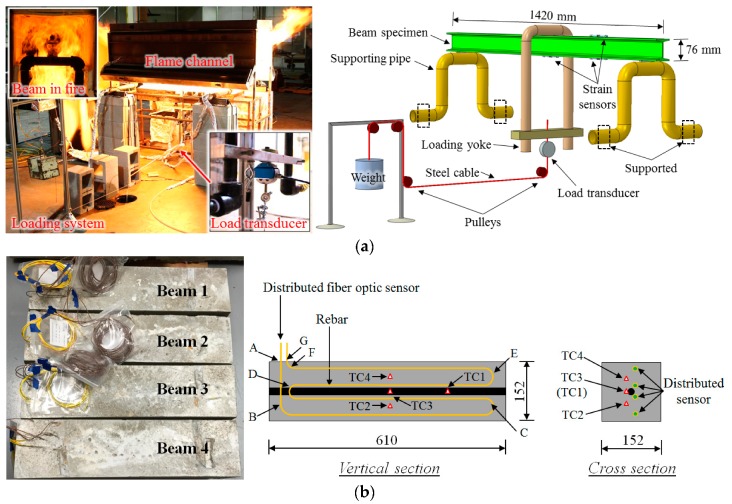
Applications of distributed fiber optic sensors in structural fire testing of: (**a**) Steel beam [125], and (**b**) reinforced concrete beam [126].

PPP-BOTDA was first applied in structural fire testing for measuring temperature and strain distributions in optical fibers installed on steel beams [67,125]. The distributed fiber optic sensors were installed along the top and bottom flanges of a hot-rolled S-shaped A36 mild steel beam exposed to the fire [125]. The steel beam was simply supported and subjected to a concentrated mechanical loading at the mid-span. Telecommunication-grade single-mode optical fibers were loosely passed through steel tubes attached on the steel beam for measuring temperature distributions, and a mechanism for scaling down the strain in the steel beam was used to design the strain sensor, as shown in Figure 11. The mechanism helps protect the optical fiber from fracture under significant deformation of the steel beam in a fire. For comparison, type-K thermocouples were used to measure temperatures, and EFPI sensors were also used to measure strains. The temperature sensor measured temperatures up to 1050 °C. However, the strain sensors failed below 600 °C, due to the increase of strain and degradation of the fused silica fiber at elevated temperatures [121]. Based on the measured temperature distributions over the beam, an enhanced thermomechanical analysis procedure was developed to predict the mechanical responses of steel beams in the fire [125]. 

Distributed fiber optic sensors were embedded in reinforced concrete beams for condition assessment [126]. Before concrete casting, telecommunication-grade single-mode optical fibers were installed in the formwork. Type-K thermocouples were embedded in the beams to measure temperatures for comparison. After the concrete cured, they were subjected to open flames from a gas burner. The distributed sensor measured temperature distributions with a 2 cm spatial resolution in the beams based on the PPP-BOTDA. Concrete cracks were identified from the measured temperature distributions. The distributed fiber optic sensors failed after significant concrete spalling occurred due to fiber fracture. 

Recently, a comparative study was conducted on measuring temperature distributions in a concrete container exposed to fire using different distributed sensing methods [127]. Fiber optic cables were attached on the surface of the container following a spiral path to map three-dimensional temperature profiles for the container. The cable had two polyimide coated optical fibers embedded inside a stainless-steel loose tube allowing fiber slipping. Temperature distribution along the optical fiber was measured using ROTDR, Rayleigh OFDR, tunable-wavelength coherent optical time domain reflectometry (TW-COTDR) based on Rayleigh scattering, PPP-BOTDA. For the ROTDR, the spatial resolution was 25 cm; the measurement duration was 60 s; the measurement distance was 100 m, and the temperature accuracy was 0.11 °C. For the Rayleigh OFDR, the spatial resolution was 3 cm, and the measurement distance was 70 m. For the TW-COTDR and PPP-BOTDA, the spatial resolution was 5 cm, and the measurement distance was 100 m. For comparison, 250 type-K thermocouples were embedded inside the concrete; 15 reference sensors were fully collocated with the fiber optic cable. While the gas temperature inside the fire chamber reached 1000 °C, the temperature of the concrete container remained below 100 °C. The discrepancy between the thermocouple and the distributed sensors was about 0.1 °C. 

## 6. Summary of Key Characteristics, Challenges, and Opportunities

### 6.1. Key Characteristics

Table 1 summarizes the fiber optic sensors discussed in this paper and their key characteristics for measuring temperature and strain in structural fire applications. The values are based on the experience of the authors and data in the cited references; they are intended as general guidance. The listed upper temperatures may not be applicable in specific cases. The sensor length refers to the length of the sensing part of point sensors or the maximum sensing distance of distributed sensors. The operating distances, spatial resolutions, and measurement times are based on typical hardware and software and may change when different hardware or software is adopted. The sensor fabrication effort and technology stage are rated to give a relative sense of the technologies. 

### 6.2. Challenges and Opportunities

A primary challenge for fiber optic sensors in structural fire engineering is the lack of reliable, effective, and efficient packaging for the optical fiber for field deployment. The packaging here refers to the means and methods used to both protect the sensor and attach it to the structure. Proper packaging is critical for both point and distributed sensors. Mechanical damage or thermal degradation of the optical fiber may cause the sensor to fail prematurely. Soot and smoke can degrade the performed of interferometer sensors. For polymer-coated optical fibers, although the fused silica fiber retains its integrity after the polymer has burned, thermal degradation may be accelerated due to the absence of the protective coating [128]. In particular, for measurements of strain in materials, reliable and well-characterized means of coupling the fiber to the structure at high temperatures are lacking; in particular for distributed fiber optic sensors.

Point sensors have shown great promise in measuring high temperature and large strains. Although point sensors have the potential to form quasi-distributed sensors through multiplexing, further research is needed to verify the feasibility in structural fire performance. The IFPI and CCMI sensors have demonstrated promising high-temperature measurement performance in the laboratory and are relatively cost-effective to fabricate. However, their structural fire performance must be evaluated in the future. Distributed fiber optic sensors have the advantage of achieving a large number of sensing points at a low sensor cost, however, the data analyzers are currently expensive and require a high level of training. 

More studies are needed for both point and distributed fiber optic sensors to characterize the uncertainty of the measurements of both strain and temperature in real structural fire conditions.

### 6.3. Other Fiber Optic Sensors for Fire Safety Study

In addition to the three types of fiber optic sensors discussed to measure temperature and strain in structural fire engineering, other fiber optic sensors have been used for fire detection by monitoring flame radiation [129,130,131], smoke [132], or gas [133,134,135] that serves as a precursor or indicator of combustion. Since different gases are produced in various types of fire, specific gas sensors must be appropriately selected. While the detection of smoke and gas indicates combustion, diffusion of smoke or gas from the combustion location to the sensors may take significant time.

Provided the availability of multiple types of sensors with different measurands, intelligent algorithms have been developed to prevent false fire alarms by combining the measurements from multiple types of sensors [136,137,138]. Furthermore, fiber optic-based fire systems can be integrated with structures’ monitoring and control system [139,140]. It is envisioned that the advancement of artificial intelligence will greatly promote the advancement of sensor technology and transform the current applications of fiber optic sensors in fire safety. 

## 7. Conclusions

Although fiber optic sensors have been widely applied for temperature and strain measurement in high-temperature applications, there remains limited knowledge of their performance in structural fire applications. This application typically has large, transient temperature variations, and challenging sensor installation conditions; typical of a building construction site. Synergized efforts of sensor designers and structural fire engineers are needed to further advance the technology. This paper reviews the sensing principles, fabrication, key characteristics, and recent applications of three classes of fiber optic sensors (i.e., grating, interferometer, and distributed sensors) in the context of structural fire engineering. The following conclusions are drawn:

- Fiber Bragg Grating (FBG) sensors that can measure temperature and strain at temperatures up to 1300 °C using fused silica fibers have been reported, however, sophisticated processes are required to achieve gratings stable at temperatures above 400 °C.
- Long-Period Fiber Grating (LPFG) that are stable up to temperatures of 800 °C have been fabricated using cost-effective and simple processes. However, LPFGs have longer sensor lengths than FBGs, resulting in great spatial averaging, and are more sensitive to bending of the optical fiber and the refractive index of the environment. It has been the author’s experience that this requires more attention to be paid to sensor installation and data interpretation.
- Fiber optic interferometer sensors have been developed to allow for the measurement of temperatures up to 1200 °C and strains up to about 10%.
- Compared with grating sensors and interferometric sensors, which are point sensors, distributed fiber optic sensors allow for the measurements of distributions along optical fibers. The upper operating temperature of distributed fiber optic sensors has exceeded 1000 °C with a centimeter-scale spatial resolution for temperature measurements. Bonding of fibers to structural steel and concrete to reliably measure strains at temperatures greater than 100 °C remains a limiting factor for distributed fiber optic sensors.
- The required measurement times for the various techniques must be considered when selecting a technology for an application.

## Figures and Tables

**Table 1:** Summary of high temperature fiber optic sensors.

Sensor	Type	Measurand	Upper Temperature	Sensor Length	Spatial Resolution	Measurement Time	Fabrication Effort	Technology Stage
Ordinary FBG	Point	T, ε	400 °C	≈5 mm	-	<1 s	Low	Mature
RFBG *	Point	T **	1295 °C	≈5 mm	-	<1 s	High	Mature
fs-FBG *	Point	T **	1100 °C	≈5 mm	-	<1 s	High	Emerging
Sapphire FBG	Point	T **	1850 °C	≈5 mm	-	<1 s	High	Emerging
LPFG	Point	T, ε	1200 °C	≈30 mm	-	<1 s	Medium	Developing
EFPI	Point	ε	1000 °C	≈5 mm	-	<1 s	High	Developing
IFPI	Point	T **	1100 °C	≈5 mm	-	<1 s	Medium	Emerging
CCMI	Point	T **	1200 °C	≈5 mm	-	<1 s	Medium	Emerging
Rayleigh OTDR	Distributed	T **	≈1000 °C	≈10 km	≈1 m	3 min	Low	Developing
Rayleigh OFDR	Distributed	T **	≈1000 °C	≈0.1 km	≈1 mm	<1 s	Low	Developing
Raman OTDR	Distributed	T **	≈1000 °C	≈30 km	≈10 mm	≈60 s	Low	Developing
Brillouin OTDA	Distributed	T, ε	≈1200 °C	≈100 km	≈20 mm	1 s–60 s	Low	Mature

* RFBG = regenerated FBG. fs-FBG = femtosecond laser fabricated FBG; ** Although the sensors are sensitive to strain, to the author’s knowledge they have not been used to measure strain in structure fires to date.
